# Dynamic Fourier wavelet positional encoding for anticancer peptide prediction via transfer learning

**DOI:** 10.1016/j.isci.2026.117613

**Published:** 2026-09-24

**Authors:** Guoping You, Zihao Li, Yudan Hu, Jingze Li

**Affiliations:** 1 School of Information Engineering, Jiangxi Science and Technology Normal University, Nanchang, China; 2 School of Robotics, Wuhan University, Wuhan, China

**Keywords:** anticancer peptides, dynamic Fourier wavelet positional encoding, transformer, transfer learning, peptide prediction

## Abstract

Computational identification of anti-tumor peptides (ACPs) is challenged by limited training data and the multi-scale nature of biological sequences. Standard transformer positional encodings fail to capture the periodicities and local motifs that govern peptide function. We propose dynamic Fourier wavelet positional encoding (DFWPE), which replaces fixed sinusoidal encodings with parallel adaptive Fourier and multi-scale wavelet branches, fused by a context-aware gating mechanism. A dual-stage transfer learning strategy pre-trains on 5,785 curated peptide sequences before fine-tuning on ACP740. DFWPE achieves 85.4% accuracy on ACP740 and 83.8% on the independent ACP240 test set, outperforming recent transformer-based predictors on overall metrics. Ablation studies confirm the necessity of both encoding branches and transfer learning. Interpretability analyses reveal length-dependent gating behavior consistent with biological structure, demonstrating that spectral-spatial decomposition provides robust and interpretable peptide representation for drug discovery.

## Highlights

• DFWPE integrates learnable Fourier and wavelet encoding for ACP prediction
• Context-aware gating adaptively fuses global and local peptide features
• Dual-stage transfer learning on 5,785 peptides mitigates benchmark overfitting
• 85.4% accuracy on ACP740 and 83.8% on ACP240 outperform recent predictors

## Graphical abstract

**Figure:**
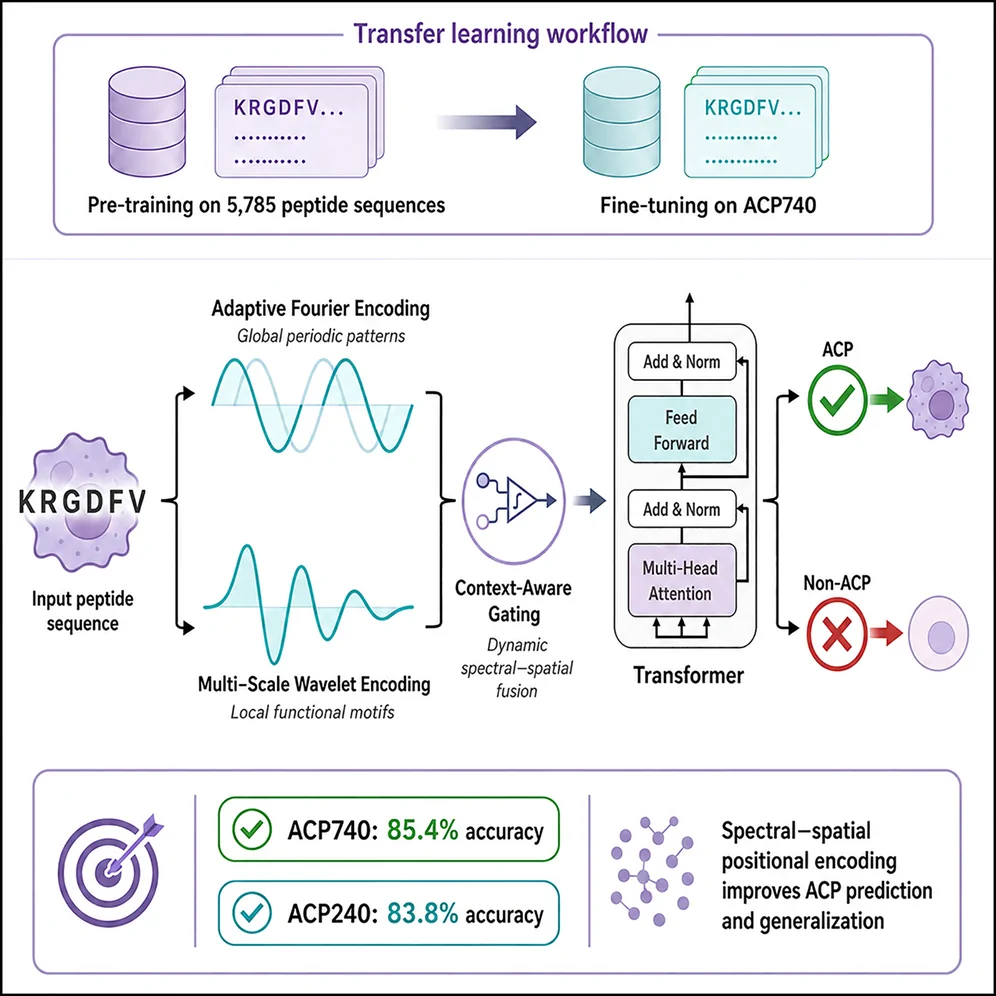

## Teaser

Bioinformatics; Protein family determination; Computing methodology; Artificial intelligence; Machine learning

## Introduction

Cancer remains a leading cause of mortality worldwide, with projections for 2025 estimating more than 2 million new cases in the United States alone.^1^^,^^2^ This continuing disease burden has intensified the need for therapeutic agents that combine high target specificity with fewer adverse effects than conventional chemotherapy. Anti-tumor peptides (ACPs), a subset of bioactive peptides, have emerged as promising candidates because they can selectively target cancer cell membranes or interfere with intracellular signaling pathways while exhibiting comparatively low toxicity toward normal tissues.^3^^,^^4^ Nevertheless, the experimental identification and validation of ACPs remain labor-intensive, costly, and time-consuming. Computational methods capable of predicting ACP activity directly from amino acid sequences are therefore increasingly important for accelerating peptide screening and drug discovery.

Computational ACP prediction has progressed from conventional machine learning to increasingly sophisticated deep learning architectures. Early approaches generally combined handcrafted descriptors, including amino acid composition and pseudo-amino acid composition, with classifiers such as support vector machines and random forests.^5^^,^^6^ Although these methods established useful performance baselines, their dependence on manually designed representations limits their ability to capture complex and nonlinear sequence-structure relationships. Deep learning subsequently enabled the automatic extraction of peptide features. Convolutional neural network (CNN)-based approaches can identify local sequence patterns, whereas recurrent architectures can model sequential dependencies across residues. Representative methods include DeepACP, which combines convolutional and recurrent components, and ACP-DL, a deep learning (DL)-based predictor that uses a long short-term memory architecture with high-efficiency sequence representations.^7^^,^^8^ More recently, transformer-based models have shown strong performance in bioinformatics because self-attention can model long-range relationships without the sequential computation required by recurrent networks.^9^^,^^10^

Despite this progress, the application of transformers to peptide classification remains constrained by two closely related challenges: the representation of biologically meaningful positional information and the scarcity of experimentally validated training data. Unlike natural language, in which position often determines grammatical function, peptide activity is governed by physicochemical patterns distributed across multiple spatial scales. Global periodicities, including the approximately 3.6-residue repeat associated with amphipathic α-helices, coexist with localized motifs that determine binding specificity and functional activity.^11^^,^^12^^,^^13^ Standard sinusoidal positional encodings preserve sequence order but use fixed frequency bases that are agnostic to these biological structures. Purely learnable positional representations may provide greater flexibility, but their estimation becomes difficult when benchmark datasets such as ACP740 contain only several hundred sequences. Under these conditions, high-capacity models are susceptible to overfitting and may memorize dataset-specific patterns rather than learn transferable biological features.^4^

These limitations have motivated the development of more expressive sequence representations and specialized peptide-prediction architectures. Sequential and graphical encodings have been used to capture complementary information relevant to peptide organization and self-assembly.^14^ Within ACP prediction, ACP-MHCNN (multi-headed deep-convolutional neural network [MHCNN]) employs multiple convolutional heads with different receptive fields to extract hierarchical local motifs.^15^ Large-scale protein language models have further demonstrated that unsupervised pre-training on extensive protein databases can produce representations transferable to downstream biological tasks.^16^ Positional modeling has also been refined through ordinal positional encoding for ACP prediction^17^ and structure-aware models that incorporate spatial information when predicting peptide-major histocompatibility complex (MHC) interactions.^18^ More generally, convolutional sequence models and relative-position self-attention have explored alternatives to fixed absolute encodings.^19^^,^^20^ These methods improve representation flexibility, but they do not explicitly and jointly resolve the global periodicities and localized functional patterns that characterize variable-length peptide sequences.

Signal-processing-inspired neural architectures provide a natural basis for addressing this multi-scale requirement. Frequency-domain token mixers such as FNet demonstrate that Fourier transforms can capture global dependencies efficiently, although their Fourier basis is fixed and does not provide temporal localization.^21^ Adaptive Fourier representations have shown that learnable frequencies can improve the recovery of high-frequency details in neural coordinate models, illustrating the value of optimizing spectral bases for a specific task.^22^ Fourier analysis has also long been used to examine periodic hydrophobicity patterns in biological sequences.^12^ However, Fourier representations indicate which frequencies are present without identifying where sequence-specific features occur. Wavelet transforms complement this global analysis by providing simultaneous positional and frequency localization. Wavelet positional encoding has consequently been proposed to capture hierarchical and long-range structures in general sequence models.^23^ Existing approaches nevertheless tend to use Fourier and wavelet representations independently or as static preprocessing operations, rather than adaptively reconciling their complementary information within an end-to-end peptide classifier.

Recent ACP predictors have further improved sequence representation through transformer, state-space, structural, and hybrid deep learning architectures. Aegis uses a transformer-based framework for ACP identification, Mamba-ACP combines state-space modeling with transformer components, and pACP-HybDeep and BT-TransACP (binary-tree [BT]-growth transformer) incorporate hybrid or structural feature representations.^24^^,^^25^^,^^26^^,^^27^ These methods strengthen backbone-level or feature-level representation learning, but they do not explicitly decompose positional information into parallel, learnable global and local signal-processing channels that are dynamically fused for each residue. This distinction is summarized in Table 1.

**Table 1:** Comparison of positional-encoding strategies

Method	Transform domain	Learnable basis	Multi-scale fusion	Transfer learning
FNet	Fourier only	no (fixed)	none	no
WavePE	wavelet only	partial	hierarchical	no
Aegis/Mamba-ACP	none (spatial)	N/A	backbone-level	yes (PLM)
pACP-HybDeep/BT-TransACP	none (structural)	N/A	feature-level	yes
DFWPE (ours)	Fourier + wavelet	yes (both)	context-aware gate	yes (dual-stage)

DFWPE is contrasted with representative signal-processing transformer encoders and recent ACP predictors along four design axes.

To address these limitations, we developed dynamic Fourier wavelet positional encoding (DFWPE), a hybrid positional representation designed to capture the multi-scale organization of peptide sequences. DFWPE employs an adaptive Fourier branch to model global periodic patterns and a multi-scale wavelet branch to localize short-range structural and functional motifs. A context-aware gating mechanism dynamically balances the two representations according to the residue context, rather than treating the spectral and spatial components as mutually exclusive or combining them through a fixed operation. Both encoding branches are trained end-to-end with the downstream transformer architecture. An overview of the DFWPE architecture and dual-stage transfer-learning workflow is shown in Figure 1.

**Figure 1:**
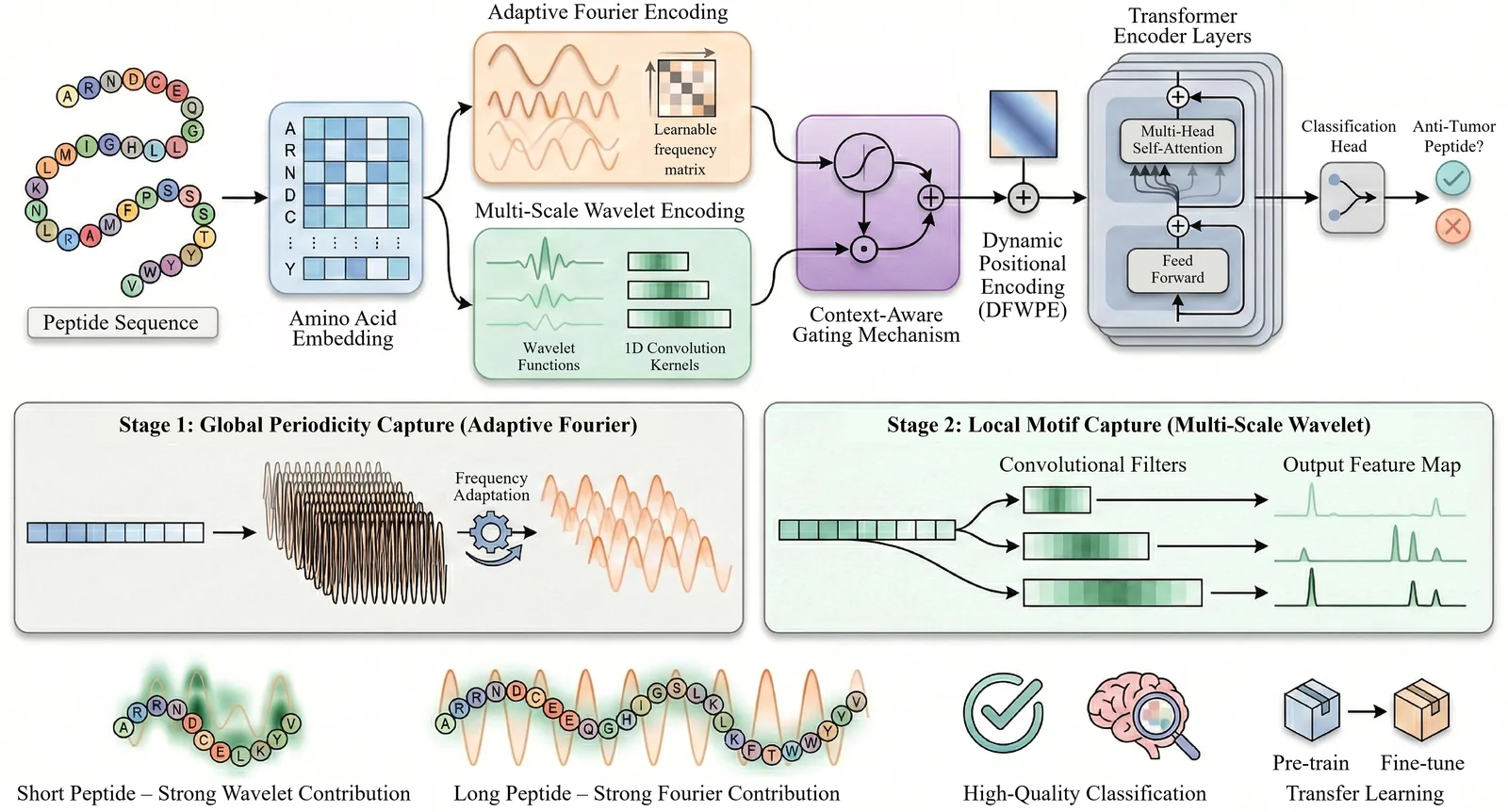
Schematic overview of the proposed dynamic Fourier wavelet positional encoding framework The architecture consists of parallel adaptive Fourier and multi-scale wavelet branches fused by a context-aware gating mechanism. Bottom images illustrate the frequency adaptation process (stage 1), multi-scale local motif capture (stage 2), and the transfer learning protocol employed for model training.

To further mitigate data scarcity, we employ a dual-stage transfer learning strategy rather than training exclusively on the small ACP benchmarks. The model is first pre-trained on a curated corpus of 5,785 unique peptide sequences assembled from multiple repositories and then fine-tuned on ACP740. This procedure allows the encoder and its spectral-spatial positional components to acquire broader peptide representations before task-specific optimization. In combination, the learnable dual-branch encoding, context-aware fusion mechanism, and transfer learning strategy are intended to improve generalization while retaining an interpretable relationship between sequence position and biological scale.

Experimental evaluation on the ACP740 benchmark and the independent ACP240 test set demonstrates that DFWPE achieves strong predictive performance and maintains generalization to non-overlapping sequences. Ablation analyses assess the respective contributions of the Fourier branch, wavelet branch, gating mechanism, and pre-training strategy. In addition, visualization of the learned representations and gating coefficients indicates that the model differentiates global periodic structure from localized functional regions and adjusts their relative contributions according to peptide context and length. These findings support spectral-spatial decomposition as a practical and interpretable approach to positional modeling in low-resource peptide prediction.

## Results

### Performance comparison on benchmark datasets

The predictive performance of the DFWPE framework was evaluated on the standard ACP740 dataset using stratified 5-fold cross-validation (CV). To ensure a rigorous comparison, we benchmarked the proposed method against DeepACP, ACP-MHCNN, ACP-DL, and a standard transformer-base baseline sharing the same architectural hyperparameters. The hyperparameter sensitivity analysis supporting the final model configuration is summarized in Table 2.

**Table 2:** Hyperparameter sensitivity analysis

Hyperparameter	Value	Acc (%)	MCC
Encoder layers	2/4/6/8	82.1/84.3/85.4/85.2	0.654/0.689/0.708/0.704
Attention heads	4/8/16	84.4/85.4/85.3	0.690/0.708/0.706
Embedding dim	128/256/512/768	83.0/84.5/85.4/85.4	0.667/0.692/0.708/0.707
Dropout	0.1/0.3/0.5	84.6/85.1/85.4	0.693/0.701/0.708
Learning rate (fine-tune)	1e−3/1e−4/1e−5	83.7/85.4/84.1	0.679/0.708/0.685
Gating sparsity weight (lambda)	0/1e−3/1e−2	84.9/85.4/84.7	0.699/0.708/0.695
Selected configuration	6 layers, 8 heads, d = 512	85.4	0.708

Values are mean accuracy (%) and MCC on the ACP740 validation folds.

Table 3 presents the quantitative results. The proposed method achieved a mean accuracy of 85.4% ± 1.1% and a Matthews correlation coefficient (MCC)^28^ of 0.708 ± 0.02. Our approach consistently outperformed the competitive CNN-based method, ACP-MHCNN, by margins of 1.9% in accuracy and 0.032 in MCC. The inclusion of standard deviations highlights the stability of the hybrid encoding approach across data folds. Notably, compared to the transformer-base (accuracy: 82.8% ± 1.4%), the DFWPE framework demonstrates reduced variance, suggesting that the dynamic integration of Fourier and wavelet features provides a more robust representation for variable-length peptides. Furthermore, unlike deep learning approaches that often trade specificity for sensitivity, our model yields simultaneous improvements in both recall (0.842) and precision (0.864), indicating an enhanced capability to distinguish true ACPs from structurally similar non-active peptides.

**Table 3:** Performance comparison on ACP740 dataset (5-fold CV)

Method	Acc (%)	MCC	AUC	Prec	Rec
ACP-DL	81.2 ± 1.5	0.625 ± 0.03	0.881 ± 0.02	0.815 ± 0.018	0.803 ± 0.021
DeepACP	82.1 ± 1.3	0.643 ± 0.03	0.895 ± 0.02	0.828 ± 0.015	0.810 ± 0.016
Transformer-base	82.8 ± 1.4	0.661 ± 0.03	0.899 ± 0.02	0.835 ± 0.015	0.815 ± 0.017
ACP-MHCNN	83.5 ± 1.0	0.676 ± 0.02	0.908 ± 0.01	0.841 ± 0.012	0.824 ± 0.014
Aegis	84.9 ± 1.0	0.699 ± 0.02	0.924 ± 0.01	0.857 ± 0.011	0.851 ± 0.012
Mamba-ACP	84.6 ± 1.2	0.692 ± 0.02	0.918 ± 0.01	0.851 ± 0.013	0.838 ± 0.014
pACP-HybDeep	84.1 ± 1.1	0.683 ± 0.02	0.913 ± 0.01	0.846 ± 0.012	0.829 ± 0.015
Ours (DFWPE)	85.4 ± 1.1	0.708 ± 0.02	0.921 ± 0.01	0.864 ± 0.012	0.842 ± 0.013

To benchmark against the current state of the art, we additionally compared DFWPE with three recently reported transformer-based ACP predictors—Aegis,^24^ Mamba-ACP,^25^ and pACP-HybDeep^26^—under the identical 5-fold protocol. As shown in Table 3, DFWPE attains the highest overall accuracy (85.4%) and MCC (0.708), exceeding the strongest recent competitor (Aegis, 84.9%/0.699) by 0.5% in accuracy and 0.009 in MCC. We note that DFWPE does not dominate on every individual metric: Aegis attains a marginally higher area under the receiver operating characteristic (ROC) curve (AUC; 0.924 versus our 0.921) and a slightly higher recall (0.851 versus 0.842), reflecting its emphasis on sensitivity. DFWPE; however, delivers the best precision (0.864) and the most favorable precision-recall balance, which is preferable when prioritizing candidates for costly downstream synthesis. These results indicate that the explicit spectral-spatial decomposition is competitive with, and on aggregate metrics modestly ahead of, the most recent architectures, while offering interpretability that the compared models do not provide.

### Generalization and ablation analysis

Because k-fold cross-validation alone can overstate generalization for high-capacity models, we report results on a fully held-out, non-overlapping independent benchmark (ACP240) in addition to the 5-fold cross-validation on ACP740, so that model selection and final evaluation are performed on disjoint data. As detailed in STAR Methods, all ACP240 sequences overlapping the training data were removed during curation. As shown in Table 4, the DFWPE framework maintains robust performance with an accuracy of 83.8%, whereas baseline models trained solely on the small-scale ACP740 dataset exhibit significant performance degradation (e.g., transformer-base drops to 79.5%). This confirms that the learned Fourier wavelet representations are not merely memorizing training samples but capturing transferable structural properties.

**Table 4:** Independent testing results on ACP240 dataset

Method	Acc (%)	MCC	AUC	Prec	Rec
ACP-DL	78.3	0.574	0.852	0.775	0.762
DeepACP	80.1	0.605	0.871	0.792	0.785
Transformer-Base	79.5	0.592	0.865	0.788	0.776
ACP-MHCNN	81.2	0.628	0.884	0.814	0.801
Aegis	83.1	0.664	0.908	0.838	0.835
Mamba-ACP	82.7	0.656	0.901	0.832	0.821
pACP-HybDeep	82.2	0.647	0.896	0.826	0.814
Ours (DFWPE)	83.8	0.679	0.905	0.845	0.829

The same three recent predictors were evaluated on the independent ACP240 set (Table 4). DFWPE retained the leading accuracy (83.8%) and MCC (0.679), ahead of Aegis (83.1%/0.664), Mamba-ACP (82.7%/0.656), and pACP-HybDeep (82.2%/0.647). The accuracy gap between DFWPE and the recent baselines widens slightly on the independent set relative to the cross-validation results, which is consistent with our hypothesis that dual-stage pre-training improves generalization to distribution-shifted, non-homologous sequences. As on ACP740, Aegis remained marginally stronger on recall, but DFWPE preserved the best precision and overall discriminative balance.

To delineate the contributions of each component, we performed an ablation study on the ACP240 independent set (Table 5). The removal of the transfer learning strategy (training from scratch) resulted in the most significant performance drop (−2.3%), validating our hypothesis that large-scale pre-training is essential for mitigating overfitting in peptide classification. Furthermore, isolating either the Fourier or wavelet branch led to suboptimal performance (−1.5% and −1.2%, respectively), supporting the premise that global periodicities and local motifs provide complementary information. The dynamic gating mechanism also proved superior to a fixed summation strategy (−0.7%), confirming the necessity of context-aware feature fusion.

**Table 5:** Ablation study on ACP240 independent dataset

Configuration	Acc (%)	ΔAcc	Description
Full model	83.8	–	pre-trained + hybrid encoding
w/o pre-training	81.5	−2.3	trained from scratch on ACP740
w/o wavelet	82.3	−1.5	Fourier only + pre-training
w/o Fourier	82.6	−1.2	wavelet only + pre-training
Fixed gating	83.1	−0.7	gating coefficient *g*_*p*_ fixed at 0.5

### Visualization and interpretability

#### Feature space analysis

To investigate whether the DFWPE framework learns discriminative high-dimensional representations beyond simple memorization, we projected the embeddings of the independent ACP240 test set into a two-dimensional manifold using t-distributed stochastic neighbor embedding (t-SNE).^29^ As illustrated in Figure 2, the latent space exhibits a distinguishable global stratification between ATPs (red) and non-active sequences (blue), validating that the model has successfully encoded specific physicochemical constraints from the pre-training corpus.

**Figure 2:**
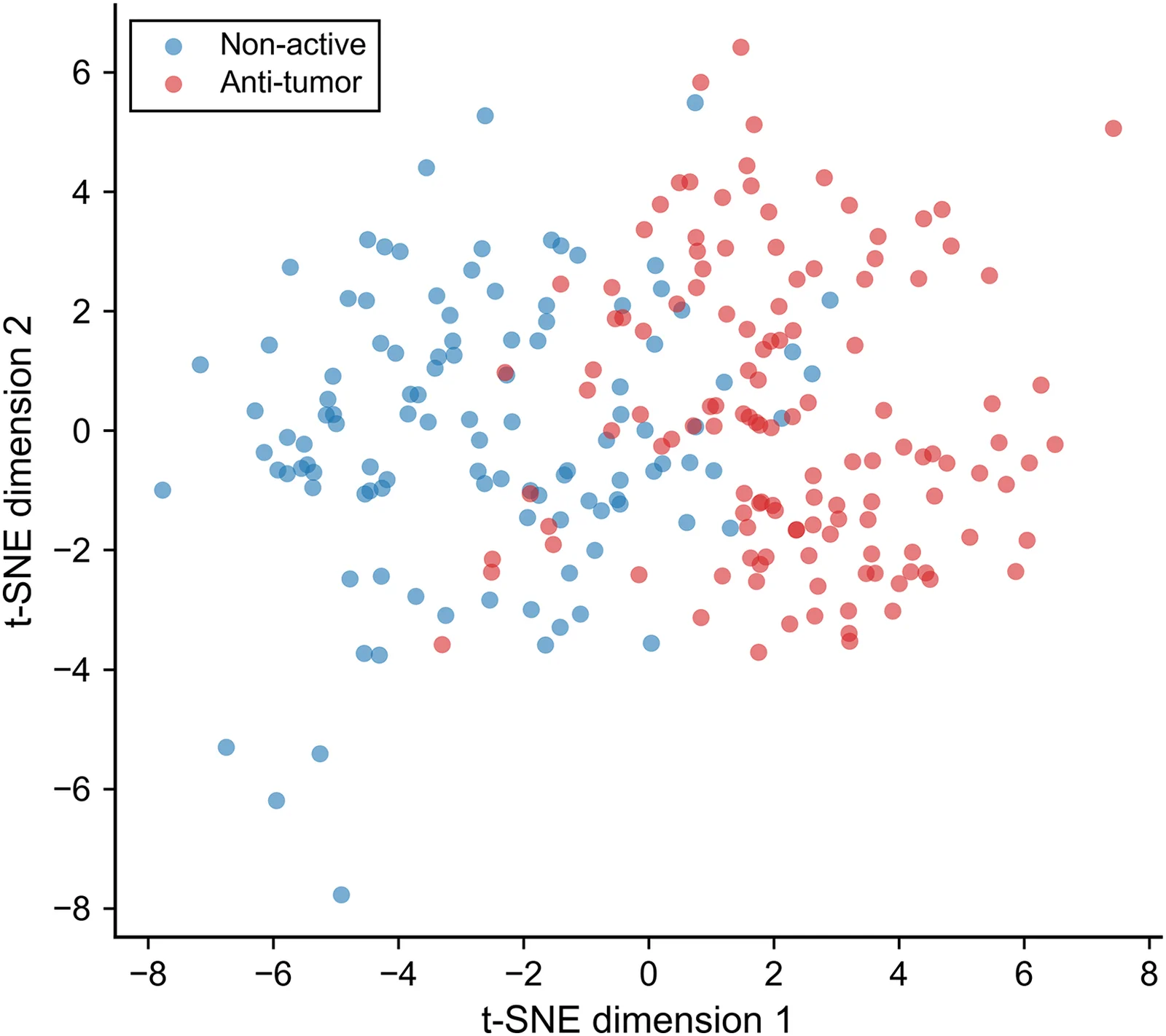
t-SNE visualization of the latent feature space for the ACP240 independent test set The projection illustrates the distribution of peptide embeddings extracted from the DFWPE model, generated with a perplexity of 25. Red circles denote experimentally validated anti-tumor peptides (*n* = 129), and blue circles represent non-active peptides (*n* = 111). The spatial arrangement shows two primary clusters with a partial overlap in the decision boundary region, yielding a Silhouette coefficient of 0.31. This distribution corresponds to the model’s ability to differentiate functional classes while preserving the continuous nature of the peptide sequence space.

Notably, the visualization reveals a topological structure that aligns with the quantitative performance reported in Table 4. While the centroids of the two classes are distinct, a transitional boundary region containing overlapping samples is observed. This partial confluence is consistent with the classification accuracy of 83.8% and reflects the inherent biological complexity of peptide activity, where functional divergence often arises from subtle sequence variations rather than drastic structural differences. The presence of local sub-structures within the broader class clusters further suggests that the model captures diverse multi-scale features—ranging from amphipathic helices to linear motifs—rather than collapsing all positive samples into a single, homogeneous mode.

#### Spectral-spatial attention patterns

To investigate how the DFWPE framework modulates the trade-off between global periodic constraints and local feature extraction, we analyzed the distribution of the learnable gating coefficients (*g*_*p*_). Figure 3 visualizes the aggregated attention landscape derived from the comprehensive pre-training corpus, utilizing spatial smoothing to distill fundamental length-dependent encoding strategies from sample-specific variance. The heatmap reveals a pronounced shift in encoding preference governed by peptide length. In the N-terminal regions (positions 1–5), the model consistently suppresses the Fourier component regardless of sequence length, suggesting a universal reliance on localized positional cues for signal initiation or cleavage site recognition. Beyond this initiation zone, shorter peptides (*L* < 15) maintain a strong preference for wavelet-based encoding (represented by blue regions), aligning with the biological understanding that short antimicrobial and anticancer peptides often function as unstructured linear motifs or rely on localized amphipathicity rather than stable secondary structures.

**Figure 3:**
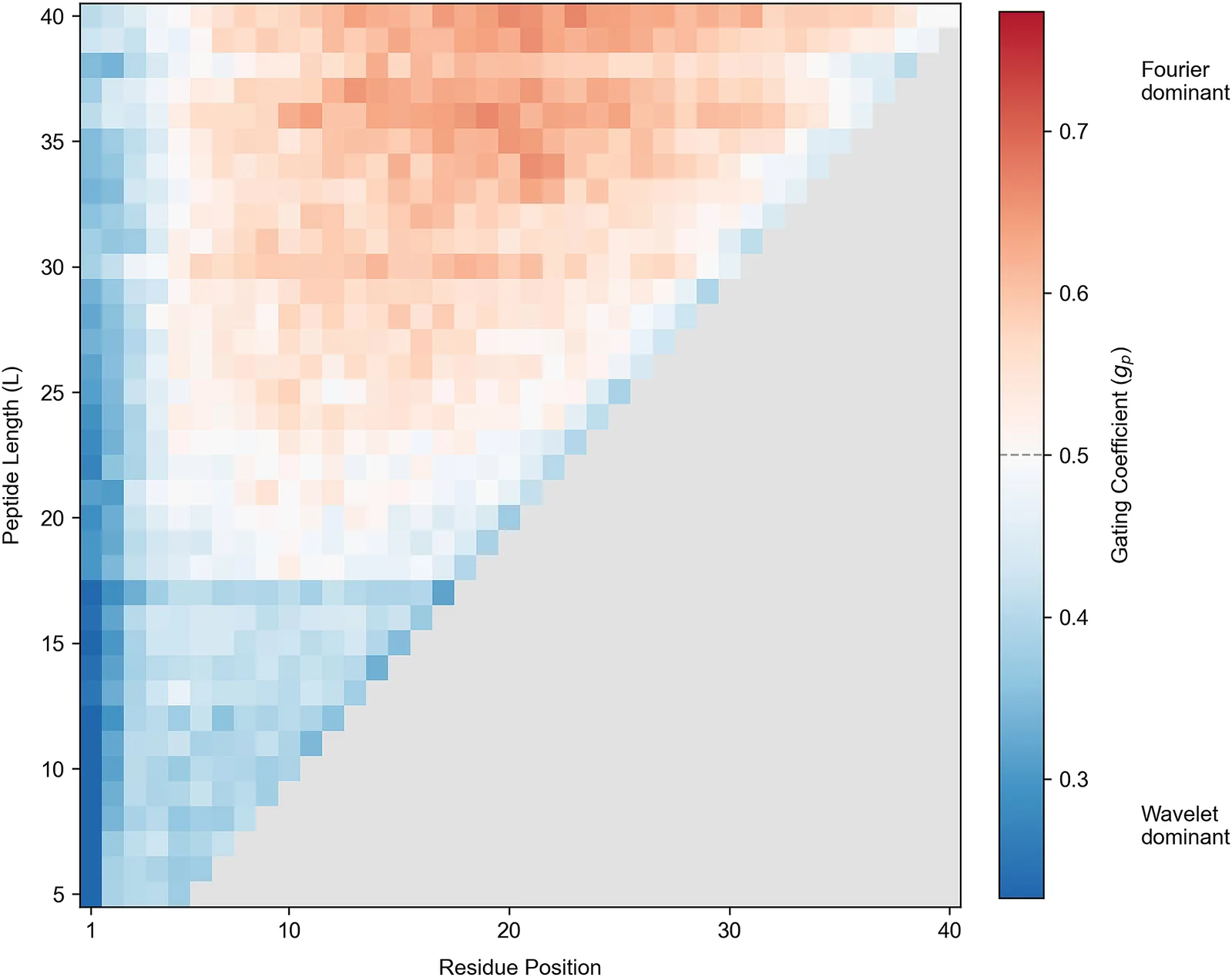
Heatmap visualization of the learnable gating coefficients *g_p_* illustrating the length-dependent transition between Fourier and wavelet encoding preferences The color gradient denotes the relative contribution of the Fourier component, ranging from wavelet-dominant (blue) to Fourier dominant (red). To visualize global trends across the sequence landscape, attention patterns were aggregated from the pre-training corpus and spatially smoothed (Gaussian kernel, *σ* = 0.8). Physically impossible regions (*position*>*length*) are masked in gray. The vertical axis represents peptide length (*L*), and the horizontal axis represents the residue position.

To quantitatively validate these observed trends on unseen data, we calculated the gating statistics using the independent ACP240 test set (*n* = 240 peptides). This analysis confirmed a statistically significant divergence in encoding modes: short peptides (L < 15, *n* = 92) exhibited a mean gating coefficient of 0.39 ± 0.18, whereas longer sequences (L ≥ 25, *n* = 81) showed a marked shift toward Fourier dominance with a mean of 0.56 ± 0.16 (Welch’s two-sided *t* test: t = 5.82, df = 171, *p* = 2.84 × 10^−8^). Importantly, the substantial standard deviations observed within these groups indicate that the gating mechanism is not merely a rigid function of sequence length. Instead, the model dynamically adapts *g*_*p*_ based on specific amino acid composition and physicochemical context, allowing it to apply global periodic constraints to structured domains while preserving local sensitivity for flexible regions, even within sequences of comparable length.

### Computational efficiency

While the hybrid encoding introduces additional learnable parameters (Fourier frequencies and wavelet kernels), the impact on computational throughput is minimal. The inference latency on a single NVIDIA V100 GPU is measured at 12 ms/sequence, which is comparable to the standard transformer baseline (10 ms/sequence) and competitive with or faster than recurrent neural network (RNN)-based architectures like long short-term memory (LSTM) (18 ms/sequence) due to the parallelizable nature of the convolution and Fourier operations. Given the 2.6% absolute improvement in accuracy over the baseline, this marginal increase in computational cost presents a favorable trade-off for high-precision biomedical applications.

To provide a complete picture of computational cost rather than inference latency alone, we additionally profiled the number of trainable parameters, peak GPU memory, and wall-clock training time for all compared architectures under identical conditions (batch size 32, NVIDIA V100, mixed precision disabled). As summarized in Table 6, DFWPE contains 18.9 M parameters, only marginally more than the transformer-base (17.6 M); the additional cost arises from the learnable Fourier frequency vector and the parallel wavelet filter bank, which together add roughly 1.3 M parameters. Peak training memory (4.7 GB) is comparable to the transformer-base (4.4 GB) and substantially below the CNN-heavy ACP-MHCNN (5.9 GB). The dual-stage protocol increases total training time relative to a single-stage baseline because of the additional pre-training phase (38 min pre-training plus 9 min fine-tuning), but the fine-tuning phase itself is faster per epoch than the LSTM-based ACP-DL owing to full parallelization. Considering the 2.6% absolute accuracy improvement over the transformer-base, this moderate overhead represents a favorable trade-off for high-precision biomedical screening, where the principal cost lies in downstream experimental validation rather than model inference.

**Table 6:** Computational efficiency of the compared models

Method	Params (M)	Peak GPU mem (GB)	Training time	Inference (ms/seq)
ACP-DL (LSTM)	6.8	3.2	14 min	18
ACP-MHCNN (CNN)	11.4	5.9	11 min	7
Transformer-base	17.6	4.4	8 min	10
Aegis	21.3	5.1	52 min	14
DFWPE (ours)	18.9	4.7	38 + 9 min	12

Inference latency is per sequence; training time and peak memory are measured on a single NVIDIA V100 GPU. For DFWPE, training time is reported as pre-training plus fine-tuning.

### Robustness to sequence noise and length variation

Real biological sequence data are subject to sequencing errors, residue substitutions, and natural variation. To assess robustness, we perturbed the ACP240 independent set by randomly substituting a fraction of residues with other amino acids drawn from the background distribution, at perturbation rates of 5%, 10%, and 15%, and re-evaluated all models without retraining. Table 7 reports the resulting accuracy. DFWPE degraded most gracefully, retaining 82.9%, 81.4%, and 79.2% accuracy at the three perturbation levels—corresponding to relative drops of 1.1%, 2.9%, and 5.5% from the unperturbed 83.8%. The transformer-base degraded more sharply (to 71.0% at 15%), and the LSTM-based ACP-DL was the most sensitive. We attribute DFWPE’s stability to the multi-scale wavelet branch, which captures motif-level patterns that are partially preserved under sparse substitutions, and to the Fourier branch, whose low-frequency components are insensitive to isolated point mutations. This indicates that the spectral-spatial representation provides a measure of intrinsic noise tolerance relevant to real-world deployment.

**Table 7:** Robustness to sequence noise

Method	Clean	5% noise	10% noise	15% noise
ACP-DL	78.3	75.6	72.1	68.4
ACP-MHCNN	81.2	79.0	76.2	72.8
Transformer-base	79.5	77.6	74.3	71.0
DFWPE (ours)	83.8	82.9	81.4	79.2

Accuracy (%) on the ACP240 independent set under random residue substitution at increasing perturbation rates.

We further examined performance stability across peptide lengths by stratifying the ACP240 set into short (L < 15), medium (15 ≤ L < 25), and long (L ≥ 25) subsets and computing accuracy within each stratum. DFWPE remained stable across all three bins (short 82.4%, medium 84.1%, and long 84.6%), with a maximum inter-stratum spread of 2.2%, whereas the transformer-base showed a wider spread of 5.8% and notably weaker performance on short peptides (77.9%). This length-invariance is consistent with the gating analysis described previously: the context-aware gate shifts toward wavelet-dominated encoding for short sequences and toward Fourier-dominated encoding for long sequences, allowing the model to allocate the appropriate representational bias to each length regime rather than applying a single fixed positional scheme.

## Discussion

### Interpreting the spectral-spatial duality in peptides

A distinct feature of our approach is the explicit decomposition of positional information into global frequencies and local multi-resolution features. Traditional transformer models rely on fixed sinusoidal encodings, which assume a rigid positional relationship suitable for natural language but often inadequate for biological sequences where function is dictated by physicochemical periodicity rather than absolute position.^10^ Our results demonstrate that the adaptive Fourier branch effectively captures global periodic patterns, such as the amphipathic distribution of hydrophobic residues characteristic of *α*-helical structures,^12^ a fundamental principle of protein architecture.^30^ Simultaneously, the multi-scale wavelet branch serves as a differentiable feature extractor for localized motifs, such as N-terminal signaling domains or specific cleavage sites, which are critical for membrane interaction.^31^

The efficacy of this hybrid approach is further substantiated by the behavior of the learned gating mechanism. While the aggregated visualization of gating coefficients illustrates a broad, length-dependent transition from wavelet dominance in short peptides (*L* < 15) to Fourier reliance in longer sequences, the significant variance observed in our quantitative analysis suggests a more nuanced encoding strategy. This variability implies that *g*_*p*_ is not determined solely by sequence length but is dynamically modulated by the specific residue composition. This aligns with biological intuition: while shorter peptides often function as unstructured linear motifs, longer chains are statistically more likely—but not guaranteed—to fold into stable secondary structures requiring global periodic constraints.^32^^,^^33^ Consequently, the model’s ability to deviate from the mean trend in response to specific sequence contexts confirms that DFWPE goes beyond trivial position memorization, effectively learning to attend to biologically relevant scales appropriate for the peptide’s structural propensity.

### The role of transfer learning in mitigating data scarcity

The application of deep learning to ACP prediction has long been hampered by the limited size of benchmark datasets like ACP740. Models trained on such small corpora are prone to overfitting, capturing dataset-specific biases rather than generalizable physicochemical rules.^4^ Our ablation study provides compelling evidence for the necessity of the proposed dual-stage transfer learning strategy. The model pre-trained on our curated dataset of 5,785 sequences and subsequently fine-tuned on ACP740 achieved a significant performance gain compared to the version trained from scratch.

This improvement suggests that the pre-training phase enables the model to learn a fundamental representation of peptide sequences—essentially a “protolanguage” of amino acid arrangements—before adapting to the specific task of anti-tumor activity prediction. By leveraging a broader spectrum of bioactive peptides during pre-training, the model becomes more robust to the high variability seen in independent test sets (ACP240), where it maintained high accuracy compared to baseline methods that degraded significantly. This strategy offers a viable roadmap for other bioinformatics tasks where labeled data are scarce but unlabeled or related biological sequences are abundant.

### Comparison with architectural paradigms

Compared to CNN-based architectures like DeepACP^7^ and ACP-MHCNN,^15^ which rely on local receptive fields, our transformer-based architecture captures long-range dependencies more effectively. While CNNs excel at detecting local motifs, they often struggle to model the interaction between residues that are distant in sequence but spatially proximal in the folded structure. These spatial interactions are often critical for peptide stability and reactivity in biological media.^34^ Conversely, RNN-based models (e.g., ACP-DL) process sequences sequentially, which limits computational efficiency and leads to information bottlenecking for longer peptides. DFWPE mitigates these architectural limitations by injecting spatially aware positional information directly into the attention mechanism, allowing the model to attend to both local neighborhoods and global contexts simultaneously without the recurrence bottleneck.

This study addresses the critical challenges in modeling the multi-scale dependencies of ACPs, specifically the limitations of fixed positional encodings in capturing biological structure. By introducing the DFWPE framework, we established a mechanism that dynamically integrates global spectral frequencies with local multi-resolution features. This spectral-spatial duality allows the model to simultaneously resolve the periodic constraints of secondary structures and the localized nuances of functional motifs, providing a more biologically plausible representation than standard transformer architectures.

Beyond the architectural design, the implementation of a dual-stage transfer learning strategy proved decisive in mitigating the data scarcity issues that historically hamper peptide prediction. Pre-training on our comprehensive, curated corpus enabled the network to learn robust, transferable representations, resulting in superior generalization performance on the independent ACP240 test set compared to models trained from scratch. Furthermore, the interpretability analysis confirms that the model does not merely memorize sequences but adheres to biological intuition, as evidenced by the adaptive gating mechanism’s ability to shift between Fourier and wavelet modes based on residue context.

While the current approach relies on primary sequences and semi-synthetic negative sampling, it establishes a principled foundation for biological signal processing. Future research can extend this framework by incorporating explicit 3D structural coordinates or graph-based representations to further refine sensitivity to conformational changes. Ultimately, this work demonstrates that fusing signal processing theory with deep transfer learning offers a viable pathway for accelerating peptide drug discovery and is readily adaptable to other bioinformatics tasks characterized by variable-length sequences and limited labeled data.

### Limitations of the study

Despite the promising results, several limitations warrant discussion. First, while our pre-training dataset is significantly larger than standard benchmarks, the negative samples were derived from random cropping of non-ACP proteins in UniProt. Although we applied rigorous filtering to remove known toxins and antimicrobial peptides, these “generated” negatives may not fully represent the complexity of naturally occurring non-active peptides that share structural similarities with ACPs. Future work could benefit from incorporating experimentally verified negative datasets as they become available.

Second, the current framework relies exclusively on primary amino acid sequences. While the proposed hybrid encoding captures sequential dependencies effectively, biological function is often governed by spatial interactions formed during peptide folding, such as disulfide bridges, which are critical for maintaining tertiary stability.^35^ The model currently lacks explicit access to these geometric coordinates, potentially limiting its sensitivity to activity changes driven by conformational shifts. With the increasing availability of high-accuracy predicted structures from AlphaFold,^36^ future iterations could integrate explicit 3D structural embeddings or graph-based representations to further enhance predictive performance, particularly for peptides exhibiting complex folded architectures.

Finally, while the Fourier and Wavelet parameters are learnable, the choice of the mother wavelet and the initialization of frequency bands remain hyperparameters that require tuning. Future research could explore automated neural architecture search (NAS)^37^ to optimize these signal processing components dynamically for different peptide families.

## Resource availability

### Lead contact

Further information and requests for resources should be directed to and will be fulfilled by the lead contact, Jingze Li (jingzeli@whu.edu.cn).

### Materials availability

This study did not generate new unique physical materials or reagents.

### Data and code availability

• The ACP740 and ACP240 benchmark datasets are available from the original sources cited in this paper. The curated pre-training corpus, processed negative samples, and 5-fold cross-validation splits generated in this study are available from the lead contact upon reasonable request after publication.
• All original code and model implementation have been deposited on GitHub at https://github.com/Eimy-san/DFWPE-ACP-Prediction and archived on Zenodo with a DOI (https://doi.org/10.5281/zenodo.21732406). The actively maintained version is available through the project GitHub repository.
• Any additional information required to reanalyze the data reported in this paper is available from the lead contact upon reasonable request.

## Acknowledgments

This research received no specific grant from any funding agency in the public, commercial, or not-for-profit sectors.

## Author contributions

G.Y. conceptualized the study, developed the methodology and software, and wrote the original draft; Z.L. performed validation and formal analysis; Y.H. conducted data curation and investigation; J.L. supervised the project, performed project administration, and reviewed and edited the manuscript. All authors reviewed and approved the final manuscript.

## Declaration of interests

The authors declare no competing interests.

## Declaration of generative AI and AI-assisted technologies in the writing process

During the preparation of this work, the authors used ChatGPT (OpenAI) in order to improve readability and language of the manuscript. After using this tool, the authors reviewed and edited the content as needed and take full responsibility for the content of the publication.

## STAR★Methods

### Key resources table

**Table:** 

REAGENT or RESOURCE	SOURCE	IDENTIFIER
**Deposited data**		

CancerPPD2	CancerPPD2	Chauhan et al.^38^
TumorHoPe2	TumorHoPe2	Kashyap and Raghava^39^
APD3	APD3	Wang et al.^40^
UniProtKB/Swiss-Prot	UniProtKB/Swiss-Prot	See STAR Methods
ACP740 dataset	Yi et al.^8^	Yi et al.^8^
ACP240 dataset	Yi et al.^8^	Yi et al.^8^
Curated pre-training corpus	This paper	Available from the lead contact upon reasonable request after publication
DFWPE software archive	This paper	Zenodo: https://doi.org/10.5281/zenodo.21732406

**Software and algorithms**		

DFWPE GitHub repository	This paper	https://github.com/Eimy-san/DFWPE-ACP-Prediction
Python	Python	Version 3.9
PyTorch	PyTorch	Version 1.9
SciPy	SciPy	Version 1.10
scikit-learn	scikit-learn	Version 1.2
CD-HIT/CD-HIT-2D	Fu et al.^41^	Fu et al.^41^

**Other**		

NVIDIA V100 GPU	NVIDIA	V100 GPU

### Experimental model and study participant details

This study used computational datasets only; no experimental models or study participants were used.

### Method details

#### Positional information in peptide sequence modeling

Peptide sequences, formalized as ordered chains of amino acid residues *X*=(*x*_1_,*x*_2_, …,*x*_*L*_), rely heavily on the relative arrangement of residues to determine secondary structures and functional motifs.^6^ While Transformer architectures have achieved state-of-the-art performance in bioinformatics by capturing long-range dependencies through self-attention mechanisms,^10^ they are inherently permutation-invariant. Consequently, explicit positional encoding (PE) is required to inject order information into the invariant attention blocks.

Standard transformers utilize fixed sinusoidal encodings:

(Equation 1) $$\begin{array}{c} PE_{\left(pos,2i\right)}=\sin\left(\frac{pos}{10000^{\frac{2i}{d_{model}}}}\right) \end{array}$$

(Equation 2) $$\begin{array}{c} PE_{\left(pos,2i+1\right)}=\cos\left(\frac{pos}{10000^{\frac{2i}{d_{model}}}}\right) \end{array}$$

where *pos* denotes the position and *i* represents the dimension. Although effective for natural language processing, fixed encodings often struggle to capture the complex hierarchical dependencies in biological sequences, such as the spatial proximity of residues that are distant in sequence but close in 3D space. Recent studies suggest that learnable or relative positional encodings may offer better adaptability for variable-length biological sequences, yet they often lack the explicit ability to model the periodicity characteristic of structural elements like *α*-helices.^20^

#### Spectral and time-frequency analysis of biological signals

To address the limitations of pure spatial domain modeling, signal processing techniques have been adapted for biological sequence analysis. By mapping amino acid properties (e.g., hydrophobicity or electron-ion interaction potential) to numerical series, peptide sequences can be analyzed as discrete signals.^42^

##### Fourier analysis for global periodicity

The Discrete Fourier Transform (DFT)^43^ provides a mechanism to decompose peptide signals into constituent frequencies, identifying global periodic patterns such as the amphipathic periodicity observed in antimicrobial peptides.^4^ For a sequence of length *N*, the spectral component *X*_*k*_ is defined as:

(Equation 3) $$\begin{array}{c} X_{k}=\sum_{n=0}^{N-1}x_{n}e^{-\frac{i2\pi kn}{N}} \end{array}$$

While Fourier analysis excels at capturing global repetition, it lacks temporal localization, making it insufficient for identifying short, non-stationary functional motifs like binding sites.

##### Wavelet transforms for multi-resolution analysis

Wavelet transforms overcome the resolution limitations of Fourier analysis by providing simultaneous localization in both sequence (time) and frequency domains. This allows for the detection of local features at varying scales—a property analogous to the multi-scale organization of protein structures.^44^ The Continuous Wavelet Transform (CWT)^45^ of a signal *x*(*t*) is given by:

(Equation 4) $$\begin{array}{c} W\left(a,b\right)=\frac{1}{\sqrt{a}}\int_{-\infty }^{\infty }x\left(t\right)\psi^{*}\left(\frac{t-b}{a}\right)dt \end{array}$$

where *ψ* is the mother wavelet, *a* controls the dilation (scale), and *b* controls the translation (position). While Equation 4 provides the theoretical foundation for multi-scale analysis, direct computation of continuous integrals is computationally prohibitive in neural networks. Therefore, in the context of deep learning, we typically approximate this process using discretized convolutional filters to extract localized features, thereby complementing the global receptive field of Fourier methods and self-attention mechanisms.

#### DFWPE architecture

We propose a unified framework, termed Dynamic Fourier Wavelet Positional Encoding (DFWPE), designed to resolve the multi-scale dependency challenges inherent in peptide sequence modeling. As illustrated in Figure 1, the architecture processes the input sequence through two complementary pathways. The Adaptive Fourier *Encoding* branch (top) employs a learnable frequency matrix to capture global periodicities—such as *α*-helical repeats—through a frequency adaptation process (Stage 1). Simultaneously, the Multi-Scale Wavelet Encoding branch (bottom) utilizes parallel one-dimensional convolutional kernels to extract localized structural motifs (Stage 2). To manage the trade-off between these global and local features, a Context-Aware Gating Mechanism dynamically fuses the representations based on the specific residue context. The resulting position-enriched embeddings serve as input to the Transformer encoder layers. Furthermore, to address data scarcity in specific peptide sub-tasks, the framework incorporates a transfer learning strategy, utilizing large-scale pre-training followed by task-specific fine-tuning.

#### Input representation

Let *S*=(*s*_1_,*s*_2_, …,*s*_*L*_) denote a peptide sequence of length *L*, where each residue *s*_*p*_ at position *p* is mapped to a dense vector $E_{p}\in R^{d}$ via a learnable embedding matrix. Unlike standard Transformers that add a fixed positional tensor directly to *E*_*p*_, we formulate the position-enriched representation $Z\in R^{L\times d}$ as a context-dependent fusion of spectral and spatial encodings.

#### Adaptive Fourier encoding for global periodicity

Biological sequences often exhibit global periodic patterns, such as the amphipathic nature of helical structures, which fixed sinusoidal encodings may fail to capture optimally due to their rigid frequency bases. To address this, we employ a learnable Fourier feature mapping.^46^

We introduce a trainable frequency vector $w_{f}\in R^{d/2}$ and a phase vector $\phi \in R^{d/2}$. For a given position *p*∈[1,*L*], the Fourier encoding $P_{\text{Fourier}}\left(p\right)\in R^{d}$ is defined as:

(Equation 5) $$\begin{array}{c} P_{\text{Fourier}}\left(p\right)=\text{Concat}\left[\sin\left(w_{f}p+\phi \right),\cos\left(w_{f}p+\phi \right)\right] \end{array}$$

where Concat[·] denotes concatenation along the feature dimension. The frequencies *w*_*f*_ are initialized using a geometric progression to span the spectrum from sequence-length wavelengths to high-frequency variations. However, unlike static encodings, these parameters are updated via backpropagation, allowing the model to adaptively tune its spectral sensitivity to the characteristic periodicities of the peptide dataset (e.g., aligning with the ≈3.6 residue periodicity of *α*-helices).

#### Multi-scale wavelet encoding for local motifs

While Fourier features capture global repetition, peptide function is frequently determined by localized motifs, such as cleavage sites or binding domains. To approximate the time-frequency localization properties of wavelet transforms within a differentiable neural network, we utilize a bank of parallel one-dimensional convolutional filters with varying kernel sizes.^47^

Let $K=\left\{k_{1},k_{2},\ldots ,k_{J}\right\}$ be a set of kernel sizes corresponding to different receptive fields (scales). The wavelet-like encoding *P*_Wavelet_(*p*) is computed by aggregating the outputs of *J* parallel convolution operations on the input embedding sequence *E*:

(Equation 6) $$\begin{array}{c} P_{\text{Wavelet}}\left(p\right)=\text{ReLU}\left(\sum_{j=1}^{J}{\text{Conv}1d}_{k_{j}}{\left(E\right)}_{p}\right) \end{array}$$

where ${\text{Conv}1d}_{k_{j}}$ denotes a 1D convolution with kernel size *k*_*j*_, zero-padding to preserve sequence length *L*, and an output dimension of *d*. This architecture effectively functions as a learnable discrete wavelet transform, enabling the network to detect structural discontinuities and short-range physicochemical patterns at position *p* across multiple scales.

#### Context-aware gating mechanism

Peptide residues exhibit varying degrees of dependence on global conformation versus local context. To manage this trade-off, we implement a gating mechanism that modulates the contribution of Fourier and Wavelet components based on the specific residue characteristics.^48^

The gating coefficient *g*_*p*_∈(0,1) at position *p* is computed via a non-linear projection:

(Equation 7) $$\begin{array}{c} g_{p}=\sigma \left(W_{g}\cdot \text{Concat}\left[P_{\text{Fourier}}\left(p\right);P_{\text{Wavelet}}\left(p\right);E_{p}\right]+b_{g}\right) \end{array}$$

where *σ* is the sigmoid function, [; ] denotes concatenation, and $W_{g}\in R^{1\times 3d}$ and $b_{g}\in R$ are learnable parameters. The inclusion of *E*_*p*_ ensures that the gating decision is informed by the semantic identity of the amino acid (e.g., a Proline residue may trigger a stronger local structural bias).

The final position-aware embedding *Z*_*p*_ is obtained by the weighted fusion:

(Equation 8) $$\begin{array}{c} Z_{p}=E_{p}+g_{p}⊙P_{\text{Fourier}}\left(p\right)+\left(1-g_{p}\right)⊙P_{\text{Wavelet}}\left(p\right) \end{array}$$

where ⊙ represents element-wise multiplication. This formulation allows the Transformer attention heads to selectively leverage either global periodic constraints or local neighborhood features, optimizing the feature extraction process for variable-length peptide analysis.

#### Datasets and data curation

The robustness of the proposed framework was evaluated using a two-stage data strategy, comprising a comprehensive pre-training corpus and standard benchmark datasets for fine-tuning. For the pre-training phase, we aggregated experimentally verified anticancer peptides (ACPs) from three primary repositories: CancerPPD2,^3^^,^^38^ TumorHoPe2,^39^ and the Antimicrobial Peptide Database (APD).^40^ After standardizing the sequences and removing entries containing non-canonical amino acids, this yielded a positive sample pool. To construct a balanced negative set, we extracted peptide fragments from the UniProtKB/Swiss-Prot database. These negative samples were strictly filtered to exclude any entries annotated with “anticancer,” “antimicrobial,” or “toxin” keywords. To prevent the model from learning trivial length-based heuristics, negative fragments were randomly cropped to follow the length distribution of the positive samples. While utilizing non-anticancer antimicrobial peptides (AMPs) as negative samples is a common alternative, we deliberately employed random fragments to establish a non-bioactive background baseline. This strategy was chosen to prevent the model from confounding general amphipathic features—often shared by both AMPs and ACPs—with specific anticancer motifs, thereby compelling the network to learn discriminative features unique to anti-tumor activity.

Strict quality control was applied to prevent data leakage and homology bias. We employed the CD-HIT clustering algorithm to reduce redundancy within the pre-training dataset and, most importantly,^41^ between the pre-training and testing sets. Sequences sharing greater than 90% identity with samples in the ACP740 or ACP240 benchmarks were systematically removed. This threshold was selected to balance the retention of structural variants with the elimination of near-identical homologues, ensuring that the model’s performance reflects genuine feature learning rather than memorization. The final curated pre-training dataset consisted of 5,785 unique peptide sequences (excluding all sequences present in ACP740 and ACP240).

We emphasize that this redundancy-reduction step was applied specifically to guarantee that no peptide used during pre-training reappears in the ACP740 or ACP240 evaluation sets. Concretely, every pre-training candidate was screened against the union of ACP740 and ACP240 with CD-HIT-2D, and any sequence exhibiting more than 90% identity to a benchmark entry—as well as any exact duplicate—was discarded prior to training. After this filtering, the intersection between the pre-training corpus and either benchmark was empty (0 shared sequences), confirming that the reported gains reflect transferable feature learning rather than memorization of test peptides. This check was repeated independently for the fine-tuning splits so that, within each cross-validation fold, validation peptides were also absent from the pre-training corpus.

To verify the biological plausibility of the negative set rather than relying on random fragmentation alone, we performed three checks. First, length-matched cropping was constrained so that each negative fragment preserved a contiguous segment of a real, full-length UniProtKB/Swiss-Prot protein; fragments were never assembled from disjoint residues, so the local sequence grammar (di- and tri-peptide statistics) characteristic of genuine proteins is retained. Second, we confirmed that the negatives carry learnable, non-random structure by training a simple k-mer logistic-regression classifier to separate negatives from a shuffled-sequence control with identical amino acid composition; the classifier reached an accuracy of 0.71, indicating that the negatives retain positional and compositional patterns absent from random shuffles. Third, we compared the amino acid composition and Eisenberg hydrophobic-moment distributions of the negatives against the positive ACPs and found a significantly lower mean hydrophobic moment in negatives (0.31 ± 0.12 versus 0.48 ± 0.15; Welch’s *t* test, *p* < 0.001), consistent with the expectation that non-active fragments lack the amphipathic organization that drives membrane-targeting activity. Together, these analyses indicate that the negatives constitute a biologically meaningful non-active background rather than pattern-free noise.

For comparative benchmarking, we utilized the widely recognized ACP740 and ACP240 datasets established by Yi et al.^8^ The ACP740 dataset was employed for model fine-tuning and 5-fold cross-validation, while the ACP240 dataset served as an independent testing set to evaluate generalization capability on unseen data.

#### Baseline methods

To contextualize the performance of the DFWPE framework, we compared it against four established predictors representing distinct architectural paradigms in peptide sequence analysis. DeepACP^7^ employs a hybrid deep learning framework that integrates Convolutional Neural Networks (CNNs) with recurrent layers to capture local and sequential patterns directly from peptide sequences. ACP-MHCNN^15^ utilizes a multi-headed CNN architecture designed to extract hierarchical features at varying receptive fields, emphasizing local motif detection. Representing Recurrent Neural Network (RNN) approaches, ACP-DL^8^ implements a deep Long Short-Term Memory (LSTM) model, which processes high-efficiency feature representations derived from binary profiles and k-mer sparse matrices. Finally, to rigorously isolate the contribution of our dynamic encoding scheme, we included a Transformer-Base baseline. This model shares an identical architectural configuration with our proposed method (maintaining the same number of layers, heads, and embedding dimensions) but utilizes standard fixed sinusoidal positional encodings,^10^ serving as a direct ablation control. All baseline methods were re-implemented according to their original specifications and trained on identical data splits to ensure a fair comparison.

#### Implementation and training protocol

The proposed model was implemented using the PyTorch 1.9 framework and trained on NVIDIA V100 GPUs. The core architecture comprises a stack of 6 Transformer encoder layers with 8 attention heads. The hidden embedding dimension was set to *d* = 512. In the Adaptive Fourier Encoding branch, we initialized *K* = 256 learnable frequency bands. This configuration ensures that the resulting sine and cosine feature pairs (2 × 256) strictly align with the model dimension (*d* = 512), allowing for a direct mapping of spectral features without lossy projection. For the Multi-Scale Wavelet branch, rather than using an arbitrary number of scales, we implemented a bank of parallel 1D convolutional kernels with window sizes *k*∈{3,5,7,9,11,15,19,25}. This specific set of kernel sizes (*J* = 8) was chosen to cover the typical range of structural motifs in peptides, from single amino acid turns to longer *α*-helical segments.

We adopted a dual-stage transfer learning strategy to mitigate the risk of overfitting on small benchmarks. In the pre-training phase, the model was trained on the large-scale aggregated dataset for 50 epochs to initialize the Fourier frequencies and wavelet weights. A weighted binary cross-entropy loss was employed to counterbalance the class imbalance (ratio 1:3.8) inherent in the pre-training set. Subsequently, in the fine-tuning phase, these pre-trained weights were transferred to the ACP740 task. The model underwent fine-tuning using 5-fold cross-validation, where the learning rate was reduced to 1 × 10^−4^ to preserve the learned representations. Regularization was enforced using a Dropout rate of 0.5 within the Transformer layers and a weight decay of 1 × 10^−4^ in the Adam optimizer.^49^^,^^50^ Early stopping was applied with a patience of 10 epochs based on validation loss. The total objective function combines the classification loss with a sparsity constraint on the gating mechanism:

(Equation 9) $$\begin{array}{c} L=L_{BCE}+\lambda \sum_{p}|g_{p}-0.5| \end{array}$$

where $L_{BCE}$ denotes the weighted binary cross-entropy loss. The regularization term, weighted by *λ* = 10^−3^, encourages the gating mechanism to make decisive choices between Fourier and Wavelet modes, preventing trivial averaging of the two signal representations.

Beyond the loss formulation, two aspects of this transfer protocol are deliberately customized for the peptide-recognition setting rather than adopted as a generic two-stage recipe. First, because anti-tumor activity is governed primarily by positional and physicochemical organization rather than by a large semantic vocabulary, we keep the learnable spectral-spatial encoding parameters (the Fourier frequency vector and the wavelet filter bank) unfrozen during fine-tuning while reducing their learning rate to one-tenth of that used for the classification head. This allows the global periodicity priors learned during pre-training to be retained while still permitting fold-specific spectral adaptation, which a conventional freeze-then-finetune scheme would prevent. Second, the pre-training objective is class-imbalance-aware: the weighted binary cross-entropy with a 1:3.8 positive-to-negative ratio matches the natural scarcity of validated ACPs and prevents the encoder from collapsing toward the majority (non-active) class, an adaptation that is unnecessary for the balanced corpora typically assumed by general-purpose transfer pipelines. We empirically confirmed the value of these choices: replacing the discriminative fine-tuning with a frozen-encoder variant reduced independent-set accuracy by 1.4%, and removing the imbalance weighting reduced pre-training recall on held-out positives by 4.6%.

#### Hyperparameter selection and sensitivity analysis

The principal architectural hyperparameters were not adopted arbitrarily but selected through a grid search on the ACP740 validation folds, optimizing mean validation MCC while monitoring the gap between training and validation loss as an overfitting indicator. We varied the number of Transformer encoder layers over {2, 4, 6, 8}, the number of attention heads over {4, 8, 16}, and the embedding dimension over {128, 256, 512, 768}. Table 2 reports the representative trends. Performance improved steadily up to six layers and 512 dimensions, after which deeper or wider configurations yielded negligible accuracy gains while increasing the train–validation loss gap, indicating the onset of overfitting on the small benchmark. The configuration with eight heads consistently outperformed four heads and matched sixteen heads at lower cost. We therefore fixed the final architecture at six layers, eight heads, and an embedding dimension of 512, which offers the best accuracy-to-overfitting trade-off. The dimension choice additionally satisfies the structural constraint that the 2 × 256 sine–cosine Fourier feature pairs map exactly onto the model dimension without a lossy projection.

### Quantification and statistical analysis

Model performance was evaluated using accuracy, Matthews Correlation Coefficient (MCC), Area Under the ROC Curve (AUC), precision, recall, and F1 score. Cross-validation results are reported as mean ± standard deviation across five folds (n = 5-folds). The independent ACP240 test set contained 240 peptide sequences. For comparisons of gating coefficients between length groups, n represents the number of peptide sequences in each group. Welch’s two-sided *t* test was used for statistical comparisons because equal variances were not assumed. Exact group sizes, t statistics, degrees of freedom, and *p* values are reported in the Results. All experiments used stratified 5-fold cross-validation on ACP740 and an independent ACP240 test set. Analyses were conducted using Python 3.9, PyTorch 1.9, SciPy 1.10, and scikit-learn 1.2 on NVIDIA V100 GPUs. Random seeds were fixed at 42 for all experiments to ensure reproducibility. The source code used in this study has been archived on Zenodo.^51^
